# The role of tumour markers in predicting skeletal metastases in breast cancer patients with equivocal bone scintigraphy

**DOI:** 10.1038/sj.bjc.6690230

**Published:** 1999-03

**Authors:** A Nicolini, P Ferrari, A Sagripanti, A Carpi

**Affiliations:** Department of Internal Medicine, Oncological consulting room, Via Roma 67, Pisa, 56126 Pisa, Italy

**Keywords:** breast cancer, bone metastases, tumour markers, equivocal bone scanning

## Abstract

Bone scintigraphy (BS) is commonly performed in the staging and postoperative monitoring of breast cancer. Nevertheless, due to low specificity it often demonstrates hot spots with equivocal interpretation, which may be misleading in the management of these patients. The aim of this study was to assess the value of a serum tumour marker panel in selecting among the patients with equivocal BS those with bone metastases. Between January 1986 and December 1995, 297 breast cancer patients were followed-up after mastectomy with serial determinations of a CEA-TPA-CA15.3 tumour marker panel, BS and liver echography. The tumour marker panel was used to select patients with equivocal BS for examination of suspicious bone areas by further imaging techniques. Up to December 1995, 158 (53%) patients showed an equivocal BS and 47 patients developed bone metastases. In the 158 patients with equivocal BS, prolonged clinical and imaging follow-up over 45 months (mean; range 12–120) was used to ascertain the presence or absence of bone metastases. In these 158 patients the negative predictive value and positive predictive value of the tumour marker panel to predict bone metastases was 97% and 75% respectively. This study shows that in breast cancer patients the CEA-TPA-CA15.3 tumour marker panel has a high value in selecting those patients with bone metastases, or at high risk of developing clinically-evident bone metastases, among the large number of subjects with equivocal BS. © 1999 Cancer Research Campaign


*British Journal of Cancer* (1999) **79**(9/10), 1443–1447
© 1999 Cancer Research Campaign
Article no. bjoc.1998.0230

### The role of tumour markers in predicting skeletal metastases in breast cancer patients with equivocal bone scintigraphy

**A Nicolini, P Ferrari, A Sagripanti and A Carpi**

Department of Internal Medicine, Oncological consulting room, University of Pisa, Via Roma 67, 56126 Pisa, Italy

**Summary** Bone scintigraphy (BS) is commonly performed in the staging and postoperative monitoring of breast cancer. Nevertheless, due to low specificity it often demonstrates hot spots with equivocal interpretation, which may be misleading in the management of these patients. The aim of this study was to assess the value of a serum tumour marker panel in selecting among the patients with equivocal BS those with bone metastases. Between January 1986 and December 1995, 297 breast cancer patients were followed-up after mastectomy with serial determinations of a CEA-TPA-CA15.3 tumour marker panel, BS and liver echography. The tumour marker panel was used to select patients with equivocal BS for examination of suspicious bone areas by further imaging techniques. Up to December 1995, 158 (53%) patients showed an equivocal BS and 47 patients developed bone metastases. In the 158 patients with equivocal BS, prolonged clinical and imaging follow-up over 45 months (mean; range 12–120) was used to ascertain the presence or absence of bone metastases. In these 158 patients the negative predictive value and positive predictive value of the tumour marker panel to predict bone metastases was 97% and 75% respectively. This study shows that in breast cancer patients the CEA-TPA-CA15.3 tumour marker panel has a high value in selecting those patients with bone metastases, or at high risk of developing clinically-evident bone metastases, among the large number of subjects with equivocal BS.

**Keywords:** breast cancer; bone metastases; tumour markers; equivocal bone scanning

The skeleton is the most frequent target for metastases in breast cancer (Meissner et al, 1971; Bonadonna et al, 1993). Many breast cancer patients are also affected by chronic degenerative, inflammatory or metabolic lesions of bone, joint or muscle (osteoarthritis, osteoporosis, myositis, fibrositis, rheumatoid arthritis, sarcoidosis, etc) due to the relatively advanced age of this population. Chronic or transient non-neoplastic bone symptomatology can mimic the metastatic bone involvement in breast cancer and vice versa. So far no humoral indicator derived from bone has proved reliable for the differential diagnosis of neoplastic bone involvement (Cuschieri, 1973; Gielen et al, 1976; Cuschieri, 1977; White et al, 1979; Mundy et al, 1984; Touitou et al, 1985). Skeletal X-rays are not a suitable tool for the 'early' diagnosis of bone metastases because they only detect lesions when the loss of calcium is at least 30–50% (Guzzo et al, 1969; Cuschieri, 1973; Gielen et al, 1976). Bone scintigraphy (BS) with $^{99m}$Tc bisphosphonates has a high sensitivity, however it also has a low specificity (Fogelman, 1991; Robinson, 1998) and it often demonstrates hot spots in those patients who remain permanently disease-free. The more recently developed diagnostic imaging techniques such as computed tomography (CT) and nuclear magnetic resonance (NMR), although helpful in the study of bony lesions (Colman et al, 1988; Nicolini et al, 1992), may not be used in all the patients with suspected metastatic disease because they are expensive and time-consuming.

*Received 18 February 1998*
*Revised 22 September 1998*
*Accepted 14 October 1998*

*Correspondence to:* A Nicolini

Thus, in breast cancer patients efforts for the diagnosis and monitoring of bone metastases lead to a great number of ineffective, expensive and potentially harmful radiological examinations. Therefore, efforts to improve the accuracy in distinguishing benign from metastatic bone involvement are of great potential utility. In addition, the 'early' detection and treatment of distant metastases is useful in prolonging disease-free interval and overall survival of some breast cancer patients (Nicolini et al, 1997).

In recent decades the serum concentration of tumour markers has been used as a warning sign of distant metastases (Nicolini et al, 1989; 1992; 1997); the combination of carcinoembryonic antigen (CEA), tissue polypeptide antigen (TPA) and breast cancer-associated antigen 115 D8/DF3 (CA 15.3) has shown 87% sensitivity in the 'early' detection of breast cancer relapses with 78% positive predictive value (Nicolini et al, 1991). Since 1986 we have adopted a protocol based upon the results of the CEA-TPA-CA15.3 tumour marker panel in the characterization of patients with equivocal BS. The aim of this study is to assess the value of a protocol based on serial determination of the serum CEA-TPA-CA15.3 tumour marker panel to identify, among the breast cancer patients with equivocal BS, those affected by bone metastases or at high risk of developing bone metastases.

### MATERIALS AND METHODS

### Patients

Between January 1986 and December 1995, 297 breast cancer patients aged 29–80 years were studied postoperatively. At postoperative histology, 116 patients showed axillary lymph-node involvement (N+) and 169 did not (N–). In the remaining 12 patients, skeletal metastases were found at the time of surgery ($M_1$). The mean follow-up time was 84 months (range 8–120).

Following mastectomy the patients were studied every 6 or 4 months according to whether they were N– or N+ at pathological examination. ER and PR were determined on tumour tissue. ER- and PR- patients were allocated to the shorter interval for follow-up. Initially the serum CEA-TPA-CA15.3 determination, routine blood tests (ESR, glucose, calcium, phosphorus, blood cell count, BUN, creatinine, GOT, GPT, gamma GT, bilirubin, alkaline phosphatase, immunoglobulins), chest X-ray, BS, liver echography and a detailed history and clinical examination were carried out to better define the postoperative staging. Baseline skeletal X-ray was performed to distinguish the benign lesions due to inflammatory and/or degenerative disease in the follow-up. Subsequently, at each visit, the CEA-TPA-CA15.3 tumour marker panel, the clinical and routine laboratory examinations were performed in addition to the history; BS and liver echography were carried out at the 24-month interval. When a relapse was suspected by tumour marker panel, BS and liver echography were performed immediately.

All invasive procedures were avoided except fine needle aspiration which was carried out at the sternum in two patients. In patients with typical metastatic or negative BS, bone metastases were considered to be diagnosed or ruled out respectively.

### Tumour markers

The TPA was measured by the Sangtec Medical (Bromma, Sweden) commercial kit. Serum levels initially > 60 mU $ml^{-1}$ and subsequently > 85 mU $ml^{-1}$ were considered elevated. Serum CA15.3 concentrations were determined by IRMA (Cis International) using a commercial kit and 32 U $ml^{-1}$ was taken as the cut-off level. CEA was measured by Lepetit Lysophase RIA (Milano, Italy) and subsequently by Sorin Biomedica (Saluggia, Italy) commercial kits; both methods gave superimposable results in appropriate comparative studies. Serum levels > 7 ng $ml^{-1}$ were considered elevated. The within and between assay coefficients of variation for CEA, TPA and CA15.3 were less than 6% and 9% respectively. When the TPA cut-off value was 60 mU $ml^{-1}$ the coefficients of variation increased to 10% and 15% respectively. In our clinical study the serum tumour marker level itself was much less important than their time-related change.

A dynamic evaluation of tumour markers was made and in cases of a high tumour marker value a further blood sample was drawn within a month of the previous elevated value. If the initial elevated tumour marker value decreased to a normal level this was considered to be an isolated elevated value. The increase in tumour marker was considered to be progressive when it was > 30% in the sample following the initial elevated value. Otherwise, two high values were regarded to be a constant elevation. Only patients with constant elevation or progressive increase in one or more tumour markers, unexplained by concomitant benign pathology and by history, were considered to be suspicious of tumour relapse (Nicolini et al, 1989; 1991; 1997).

### Bone scintigraphy

BS was performed 2–3 h after injection of 15–20 mCi $^{99m}Tc$ hydroxymethyldiphosphonate (HMDP) and bone imaging was performed initially with an analogue and then a digital whole-body gamma camera (models SELO KR7 and General Electrics STARCAM 400 or 3000 respectively). In order to better interpret the BS findings, the history and benign lesions (osteoarthritis, osteoporosis, trauma, etc) detected with baseline skeletal X-ray were taken into account. The principal criteria for interpretation of the BS and the principal types of BS patterns were as follows.

- Findings very likely due to bone metastases (pathological BS): multiple obvious hot spots in sites that are usual for breast cancer metastases and not involved by benign lesions.
- Findings very likely not due to bone metastases (negative BS): no hot spot; one or more hot spots with diffuse irregular uptake; one or more hot spots in sites that are unusual for breast cancer metastases and involved by benign lesions.
- Findings defined as equivocal for bone metastases (equivocal BS): slight asymmetry of tracer uptake; one or more hot spots in sites that are unusual for breast cancer metastases (skull, arms) and not involved by benign lesions; one or more hot spots in sites that are usual for breast cancer metastases also involved by benign lesions; a single hot spot in sites that are usual for breast cancer metastases and not involved by benign lesions.

### Management of patients with equivocal BS

Patients with equivocal BS and concomitant constant elevation or progressive increase in one or more tumour markers were selected for radiological examinations. Constant elevation or progressive increase in tumour markers were defined as mentioned above. All hot spots on the BS with an equivocal interpretation and selected for radiological examination were examined by CT, except those in the ribs that were evaluated by skeletal X-ray. Skeletal X-ray and CT were carried out according to standard techniques. Specifically, for CT performed using a CT SYTEC 3000 GE, slice thickness was 1 mm, spacing 1 mm and scan time 2 s, and for a CT 9800 QUICK, high resolution, slice thickness 1.5 mm, spacing 3 mm and scan time 2 s.

### Statistical analysis

Probably positive tests were defined as: (a) radiological examination (i.e. baseline skeletal X-ray, skeletal X-ray and CT directed to the hot spots of BS) that was pathological or baseline skeletal X-ray that showed a picture of equivocal interpretation and (b) tumour marker assay panel with constant elevation or progressive increase in one or more antigens, unexplained by concomitant transient or chronic benign pathology. Pathological BS and those showing a picture of equivocal interpretation were also considered probably positive tests. Probably positive tests which were confirmed by monitoring to death or by a definite clinical-imaging course after the initial result were defined as true positives.

Probably negative tests were defined as: (a) negative radiological examination and negative BS and (b) CT without definitely abnormal result and (c) tumour marker assay panel with normal or isolated elevated value that persisted longer than 6 months after the first abnormal result. Probably negative tests which were followed by at least 1 year of survival without any clinical-imaging signs of relapse were evaluated as true negatives.

In patients where CT and/or skeletal X-ray were directed to multiple BS areas with equivocal interpretation, a single test result

 © Cancer Research Campaign 1999

was computed. The overall result was classified as negative when it was negative in all uncertain BS areas, equivocal or pathological when it was uncertain or pathological in at least one of the BS areas with equivocal interpretation. The final result was related to the most relevant of the equivocal BS areas evaluated by CT and/or skeletal X-ray.

Sensitivity was defined as TP/(TP + FN) × 100, specificity as TN/(TN + FP) × 100, accuracy as TN + TP/(TN + FN + TP + FP), positive predictive value as TP/(TP + FP), negative predictive value as TN/(TN + FN), where FP = false positive, FN = false negative, TP = true positive, TN = true negative.

In patients with a BS with equivocal interpretation the exact Fisher's test was applied to verify whether tumour marker panel had statistically significant power in distinguishing true positives from true negatives.

### RESULTS

### General outcome and BS with equivocal interpretation

Up to December 1995, 47 (16%) of the 297 patients manifested distant metastases and in 35 of them these occurred during the postoperative follow-up. Twenty five of these 35 patients were N+ and the remaining 10 N–. In 42 of the 47 patients with metastases, bone was the first site of distant spread. In the other five, various organs were involved contemporaneously with bone.

During the postoperative follow-up 158 (53%) of the 297 patients showed hot spots on BS with equivocal interpretation. One hundred and thirty-six of them (86%) (group a) were in the 250 non-relapsed patients and the remaining 22 (14%) (group b) were in the group of 47 with bone metastases. Twenty-four (15%) of these 158 patients, six (4%) in group a (non-relapsed) and 18 (82%) in group b (bone metastases), were selected on the basis of constant elevation or progressive increase in one or more of tumour marker panel to undergo CT or skeletal X-ray directed towards BS hot spots with equivocal interpretation.

### Non-relapsed patients

Table 1 shows the principal results of tests performed in the 250 non-relapsed patients. In these patients the overall specificities for excluding bone metastases of baseline skeletal X-ray, BS and tumour marker panel were 97%, 46% and 99% respectively.

One hundred and thirty-six (54%) (group a) of these non-relapsed patients had hot spots on BS with equivocal interpretation. In that group a, aged 33–80 years (57 ± 12; mean ± SD), 32% of patients were premenopausal and 68% were N+, and bone metastases were not confirmed during a mean clinical-imaging follow-up over more than 47 months (range 12–120). Six (4%) of these 136 non-relapsed patients with equivocal BS were selected to undergo CT because of concomitant constant elevation or progressive increase on tumour marker panel.

**Table 1** False positive results in diagnosing bone metastases in 250 nonrelapsed breast cancer patients

| Test type | T*n* | Result | Probably positive tests (*n*) | Test specificity (%) |
|---|---|---|---|---|
| Tumour marker panel | 3792 | 94+ | 2 | 99 |
| Bone scintigraphy | 895 | 136e | 136 | 46 |
| Skeletal X-ray | 250 | 7e | 7 | 97 |

T*n* = total number of tests; Result = number of patients with (e) equivocal initial imaging result or (+) significantly elevated tumour marker panel

### Relapsed patients

Table 2 shows the principal results of tests performed in the 47 relapsed patients. In these 47 relapsed patients the overall sensitivities for diagnosing bone metastases of baseline skeletal X-ray, BS and tumour marker panel were 25%, 98% and 91.5% respectively.

In 14 (29.7%) equivocal (seven) or pathological (seven) BS was the first sign of the relapse. Twenty-seven patients (57%) were suspected for relapse because they showed constant elevation or progressive increase in one or more tumour markers in the panel, which was not explained by concomitant chronic or transient benign pathology. In 15 of these 27 patients, BS was pathological, and in the other 12 patients it showed a picture of equivocal interpretation. In the other two (4.2%) of the 47 relapsed patients, baseline skeletal X-ray was the first sign of the relapse. In one of these two patients BS was pathological and in the other it was falsely negative. Bone symptomatology occurred as a first sign of relapse in the remaining four (8.5%) patients and lead-time to definite radiological diagnosis was 5.5 ± 7 months. In three of these four patients BS showed a picture of equivocal interpretation and in the last it was pathological. In 23 relapsed patients bone symptoms appeared either after patients were suspected due to tumour marker increase (14 subjects) or when bone metastases were ascertained by radiological means (nine patients). In the remaining 20 relapsed patients bone symptoms occurred after bone metastases had been radiologically ascertained. Therefore, at the time of relapse in 24 (51%) of the 47 patients with metastases the BS was pathological.

**Table 2** False negative results in 47 breast cancer patients with bone metastases

| Test type | T*n* | Result | Probably negative tests (*n*) | Test sensitivity (%) |
|---|---|---|---|---|
| Tumour marker panel | 578 | 20 – | 4 | 91.5 |
| Bone scintigraphy | 104 | 22 e | | 98 |
| | | 1 negative | 1 | |
| Skeletal X-ray* | 47 | 15 negative | 15 | 25 |

T*n* = total number of tests; Result = number of patients with (e) equivocal initial imaging result or (–) normal or not significantly elevated tumour marker panel; *basal skeletal X-ray was evaluated in 20 patients with bone metastases in the first year after mastectomy

© Cancer Research Campaign 1999 

**Table 3** Tumour marker panel in patients with equivocal bone scintigraphy (BS)

| | Patients without bone metastases | Patients with bone metastases |
|---|---|---|
| Equivocal BS (*n*) | 136 | 22 |
| Tumour marker panel + (*n*) | 6 | 18 |
| Tumour marker panel – (*n*) | 130 | 4 |
| Tumour marker panel specificity | 96% | – |
| Tumour marker panel sensitivity | – | 82% |

(+) significantly elevated; (–) normal or not significantly elevated tumour marker panel; *P* = 0.02, exact Fisher's test

In 22 (46.8%) it showed a picture of equivocal interpretation and in one patient it was falsely negative. In this last patient (aged 53 years) with $pT_2N_1M_0$ classification, BS maintained the same picture until 33 months after the first falsely negative result. This patient had an osteolytic lesion in the occipital bone which was suspected to be a relapse because of the tumour marker panel and was submitted to CT of the skull because of constant headache.

Twenty two (47%) (group b) of the 47 relapsed patients showed a BS with an equivocal picture (Table 2). In this group b, aged 32–77 years (53.5 ± 12; mean ± SD), premenopausal and N+ patients were 41% and 59% respectively, and bone metastases were confirmed by an average clinical-imaging follow-up over more than 28 months (range 5–65). Eighteen (82%) of these 22 relapsed patients with equivocal BS showed constant elevation or progressive increase on the tumour marker panel (Table 3) and were selected to undergo CT (14) or rib X-ray (four).

### The role of tumour markers in confirming or excluding bone metastases in the patients with equivocal BS

Table 3 clearly shows that tumour markers were elevated among relapsed patients in a significantly greater proportion than in non-relapsed patients. Tumour markers remained normal or not significantly elevated in patients without bone metastases much more frequently than in those who relapsed (*P* = 0.02, Fisher's exact test).

### DISCUSSION

The physician who cares for breast cancer patients after mastectomy very often faces the clinical problem of how to distinguish between benign and metastatic bone lesions without using invasive procedures. He should therefore be well aware of the accuracy of the available techniques.

This study shows that the sensitivity of baseline skeletal X-ray for the 'early' detection of bone metastases is only 25%. BS is a useful sensitive tool for the early diagnosis of bone metastases but is not specific enough. Furthermore, in this study, it provided a picture of equivocal interpretation in 53% of all patients (54% of non-relapsed patients and 47% of patients with bone metastases).

Therefore, skeletal X-ray is not useful for the 'early' detection of bone metastases due to low sensitivity (Galasko et al, 1972; Komaki et al, 1979) and BS requires complementary tools, to select patients to undergo radiological examinations directed to those hot spots with equivocal interpretation. NMR and CT are among the more recently developed techniques. NMR has been reported to be very helpful for the 'early' detection of bone marrow microdissemination from breast cancer. It allows for a better staging; however, the diagnostic information provided is limited to the regions of haemopoietic bone marrow (Sanal et al, 1994; Moulopulos et al, 1995). In contrast, hot spots on whole body BS commonly occur in compact portions of the bony skeleton. CT has proved highly accurate in the diagnosis of bone lesions (Held et al, 1994). However CT and NMR may not be suitable for routine use in the postoperative follow-up of breast cancer patients because they are too expensive and time-consuming; furthermore, the lesions examined are often benign. The sensitivity of the CEA-TPA-CA15.3 panel to signal a relapse is very high (about 90%). This high sensitivity for detecting distant metastases confirms our previous data (Nicolini et al, 1991) and it is greater than that reported by other authors for individual markers (50%, 67%, 77% for CEA, CA15.3, TPA respectively) and for the combination of CEA-CA15.3 (71%) (Luthgens et al, 1981; Fateh-Moghadam et al, 1993; ASCO tumour marker expert panel, 1996). The reason for this greater sensitivity is probably because three markers are monitored simultaneously (CEA, TPA and CA15.3): Moreover, we have defined specific criteria to seek out false positive results on the tumour marker panel (Nicolini et al, 1989, 1992).

In this study, we evaluated the utility of the CEA-TPA-CA15.3 tumour marker panel to select patients with equivocal BS at high risk of bone metastases. In selecting patients with equivocal BS to undergo radiological examinations, the CEA-TPA-CA15.3 tumour marker panel showed 94% accuracy. In fact, among the 158 patients with equivocal BS (groups a + b) only four falsely negative and six falsely positive results of the tumour marker panel were observed (Table 3).

These data indicate that in the postoperative follow-up of breast cancer patients the CEA-TPA-CA15.3 tumour marker panel is a suitable tool to select patients to undergo further radiological examinations directed at the sites of equivocal BS findings and that it allows a decrease of 70–80% in the number of bone scintigraphic studies and other radiological examinations (chest and skeletal X-ray, liver echography, CT, NMR). Consequently, a 50–75% cost saving per patient is achieved for an intensive 5-year follow-up with CEA-TPA-CA15.3 tumour marker panel, compared with that conducted with traditional means. This cost has been calculated as $10 000 per patient (Virgo, 1996) while in our protocol it amounted to $2500.

As to the differential diagnosis between benign and malignant skeletal lesions. CT showed high accuracy (70%) while skeletal X-ray directed to equivocal hot spots shown at ribs by BS proved unhelpful. Different examinations such as spiral CT and FDG PET

 © Cancer Research Campaign 1999

(Shreve et al, 1996) should be evaluated for the differential diagnosis of equivocal BS areas in the ribs.

In conclusion, CEA-TPA-CA15.3 tumour marker panel can be used as a preliminary screen to select those who need further radiological investigation, thus confirming its important role in the postoperative follow-up of breast cancer patients (Nicolini et al, 1997).

### REFERENCES

ASCO Tumor Marker Expert Panel (1996) Clinical practice guidelines for the use of tumor markers in breast and colorectal cancer. *J Clin Oncol* **14**: 2843–2877

Bonadonna G and Valagussa P (1993) Neoplasie della mammella. In *Manuale di oncologia medica*, Bonadonna G and Robustelli della Cuna G (eds), pp 529–563. Masson: Milano

Colman LK, Porter BA, Redmond J III, Olson DO, Stimac GK, Dunning DM and Friedl KE (1988) Early diagnosis of spinal metastases by CT and MR studies. *J Comput Assist Tomogr* **12**: 423–426

Cuschieri A (1973) Urinary hydroxyproline excretion in early and advanced breast cancer – a sequential study. *Brit J Surg* **60**: 800–803

Cuschieri A (1977) Urinary hydroxyproline in the management of breast cancer. *World J Surg* **1**: 299–302

Del Turco MR, Palli D, Cariddi A, Ciatto S, Pacini P and Distante V (1994) Intensive diagnostic follow-up after treatment of primary breast cancer. A randomized trial. *JAMA* **271**: 1593–1597

Fateh-Moghadam A and Stieber P (1993) Use of tumour markers in certain solid tumours. In *Sensible use of tumour markers*, Fateh-Moghadam A, Stieber P (eds), pp 56–57. J Hartmann, Verlag GMBH: Munich

Fogelman I (1991) Bone scanning. In *Clinical Nuclear Medicine*, 2nd edn, Maisey MN, Britton KE and Gilday DL (eds), pp 131–157. Chapman and Hall Medical: London

Galasko CSB and Doyle FH (1972) The detection of skeletal metastases from mammary carcinoma. A regional comparison between radiology and scintigraphy. *Clin Radiol* **23**: 295–297

Gielen F, Dequeker J, Drochmans A, Wildiers J and Merlevede M (1976) Relevance of hydroxyproline excretion to bone metastasis in breast cancer. *Br J Cancer* **34**: 279–285

Guzzo CE, Pachas WN, Pinals RS and Krant MJ (1969) Urinary hydroxyproline excretion in patients with cancer. *Cancer* **24**: 382–387

Held P and Breit A (1994) Comparison of CT and MRI methods in diagnosis of tumours of the para- and retropharingeal space and temporal bone. *Bildgebung* **6**: 263–271

Komaki R, Donegan W, Manoli R and Yeh EL (1979) Prognostic value of pretreatment bone scans in breast carcinoma. *AJR* **132**: 877–881

Luthgens M, Schelegel G, Eklund G and Bjorklund B (1981) Correlation between activity in breast cancer and CEA, TPA and eighteen common laboratory procedures and the improvement by the combined use of CEA and TPA. *Tumour Diagnostik* **2**: 6–11

Meissner W and Warren S (1971) Neoplasms. In *Pathology*, 6th edn. Mosby: St. Louis

Moulopulos LA, Dimopoulos MA, Smith TL, Weber DM, Delasalle KB, Libshitz HI and Alexanian R (1995) Prognostic significance of magnetic resonance imaging in patients with asymptomatic multiple myeloma. *J Clin Oncol* **13**: 251–256

Mundy GR, Ibbotson KJ, D'Souza SM, Simpson EL, Jacobs JW and Martin TJ (1984) The hypercalcemia of cancer-clinical implications and pathogenic mechanisms. *N Engl J Med* **310**: 1718–1722

Nicolini A, Carpi A, Di Marco G, Giuliani L, Giordani R and Palla S (1989) A rational postoperative follow-up with carcinoembryonic antigen, tissue polypeptide antigen and urinary hydroxyproline in breast cancer patients. *Cancer* **63**: 2037–2046

Nicolini A, Colombini C, Luciani L, Carpi A and Giuliani L (1991) Evaluation of serum CA 15-3 determination with CEA and TPA in the post-operative follow-up of breast cancer patients. *Br J Cancer* **64**: 154–158

Nicolini A, Carpi A and Tibaldi C (1992) The patients postoperative management of breast cancer patient: new concepts. In *Progress in Clinical Oncology*, Carpi A, Sagripanti A and Mittermayer CH (eds), pp 187–203. Sympomed Medical: Munchen

Nicolini A, Anselmi L, Michelassi C and Carpi A (1997) Prolonged survival by "early" salvage treatment of breast cancer patients: a retrospective six year study. *Br J Cancer* **76**: 1106–1111

Robinson LA (1997) Radioisotope-guided surgical biopsy of suspected osseous metastases. *Cancer Control* **4**: 517–522

Sanal SM, Flickinger FW, Caudell MJ and Sherry RM (1994) Detection of bone marrow involvement in breast cancer with magnetic resonance imaging. *J Clin Oncol* **12**: 1415–1421

Shreve PD, Grossman HB, Gross MD and Wahl RL (1996) Metastatic prostate cancer: initial findings of PET with 2-deoxy-2-(F-18) fluoro-D-glucose. *Radiology* **199**: 751–756

Touitou Y, Proust J, Klinger E and Nakache JP (1985) Biological and pathological factors affecting plasma gamma-glutamyl transpeptidase and alkaline phosphatase activity in the elderly. *Clin Chim Acta* **146**: 1–10

Virgo KS (1996) The costs of cancer patient follow-up post-treatment. In: ASCO Educational Book, pp 328–329. the American Society of Clinical Oncology: Place

White DR, Maloney JJ III, Muss HB, Vance RP, Barnes P, Howard V, Rhyne L and Cowan RJ (1979) Serum alkaline phosphatase determination-value in the staging of advanced breast cancer. *JAMA* **242**: 1147–1149

*© Cancer Research Campaign 1999*
